# Characterization of Nanoscale Organization of F-Actin in Morphologically Distinct Dendritic Spines *In Vitro* Using Supervised Learning

**DOI:** 10.1523/ENEURO.0425-18.2019

**Published:** 2019-08-01

**Authors:** Siddharth Nanguneri, R. T. Pramod, Nadia Efimova, Debajyoti Das, Mini Jose, Tatyana Svitkina, Deepak Nair

**Affiliations:** 1 Centre for Neuroscience, Indian Institute of Science, Bangalore, 560012, India; 2 Department of Biology, University of Pennsylvania, Philadelphia, PA 19104

**Keywords:** Alzheimer’s disease, dendritic spines, F-actin, segmentation and pattern recognition, super-resolution, supervised learning

## Abstract

The cytoarchitecture of a neuron is very important in defining morphology and ultrastructure. Although there is a wealth of information on the molecular components that make and regulate these ultrastructures, there is a dearth of understanding of how these changes occur or how they affect neurons in health and disease. Recent advances in nanoscale imaging which resolve cellular structures at the scale of tens of nanometers below the limit of diffraction enable us to understand these structures in fine detail. However, automated analysis of these images is still in its infancy. Towards this goal, attempts have been made to automate the detection and analysis of the cytoskeletal organization of microtubules. To date, evaluation of the nanoscale organization of filamentous actin (F-actin) in neuronal compartments remains challenging. Here, we present an objective paradigm for analysis which adopts supervised learning of nanoscale images of F-actin network in excitatory synapses, obtained by single molecule based super-resolution light microscopy. We have used the proposed analysis to understand the heterogeneity in the organization of F-actin in dendritic spines of primary neuronal cultures from rodents. Our results were validated using ultrastructural data obtained from platinum replica electron microscopy (PREM). The automated analysis approach was used to differentiate the heterogeneity in the nanoscale organization of F-actin in primary neuronal cultures from wild-type (WT) and a transgenic mouse model of Alzheimer’s disease (APP_Swe_/PS1ΔE9).

## Visual Abstract

**Figure:**
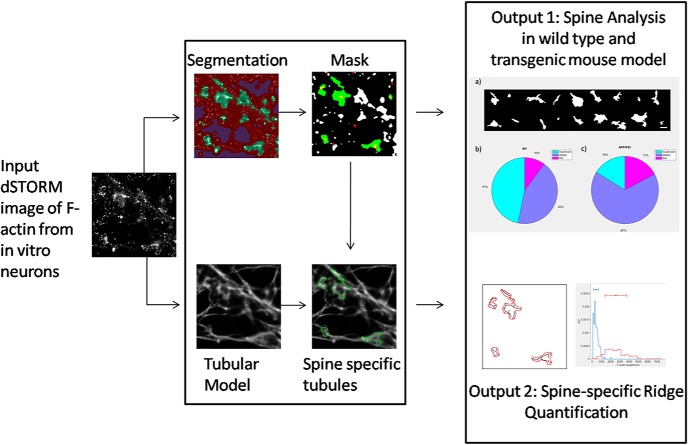

## Significance Statement

Organization of F-actin in dendritic spines is known to be important in maintaining the structure and function of excitatory synapses. Multicolor super-resolution microscopy enables us to have better insights into its organization in health and disease. Here, we have combined novel methods for the analysis of nanoscale images of F-actin network using segmentation with pattern recognition based on supervised learning. This automated approach was validated using platinum replica electron microscopy (PREM) images of F-actin organization in dendritic spines. Furthermore, we have explored the differences in the nanoscale F-actin network in wild-type (WT) and transgenic mouse models of Alzheimer’s disease using this novel approach.

## Introduction

Dendritic spines in neurons are important structures that mediate neuron to neuron communication. The morphology and molecular composition of spines determine the efficacy of signal transmission. The morphologic changes during transmission are accompanied by an alteration in the composition of molecules, and thus the relative strength of the synapses. The filamentous form of the cytoskeletal molecule actin (F-actin) is a morphologic and functional determinant of individual spines (Hotulainen and Hoogenraad, 2010). The advent of high resolution microscopy techniques has revealed the assembly and architecture of F-actin in various subcompartments of neurons (Frost et al., 2010; Urban et al., 2011; Chazeau et al., 2014; Efimova et al., 2017). The recent observations of actin rings have also highlighted the heterogeneity of F-actin organization in neuronal processes (Xu et al., 2013). Although electron microscopy studies have shown the distribution of F-actin inside spines, very few attempts have been made to evaluate the F-actin organization using super-resolution light microscopy. Recent studies have indicated that F-actin in spines can be organized as outwardly radiating rods, and the organization of these rods can be affected very early during the onset of Alzheimer’s disease (Kommaddi et al., 2018). However, high throughput and objective analysis to classify the synaptic actin cytoskeleton, derived from super-resolution imaging, is still missing.

Platinum replica electron microscopy (PREM) has been instrumental in providing high resolution images of the actin cytoskeleton in dendritic spines. Thin filamentous structures, whose diameter fits that of F-actin, form the predominant cytoskeleton of the spine (Efimova et al., 2017). Using light microscopy, most of the morphologic changes in the spine have been studied indirectly with the help of volume markers such as GFP or dextran conjugated dyes (Mancuso et al., 2013). Conjugating dyes to proteins of interest or creating fusion constructs can create undesirable effects due to excessive expression and steric interference with protein functions (Ansari et al., 2016). Alternatively, there have been advances in identifying chemical probes which can bind to F-actin, thus enabling a direct read-out of the F-actin architecture from different subcellular compartments (Lukinavicius et al., 2014; Nanguneri et al., 2014). Thus, it is feasible for these probes to be used with regular immunocytochemistry along with other molecules to comprehend the fine organization of F-actin in different neuronal compartments. With a rising interest in investigating the role of F-actin morphology and spine compartmentalization in neurodegenerative diseases, it is essential to develop approaches that enable direct probing of F-actin assembly in spines (Bamburg and Bernstein, 2016; Androuin et al., 2018; Kommaddi et al., 2018).

In this paper, a novel approach for the analysis of F-actin network in dendritic spines is presented using data from super-resolution light microscopy, namely direct stochastic optical reconstruction microscopy (dSTORM; Heilemann et al., 2008), in combination with an analytical method called super-resolution by radial fluctuations (SRRF). SRRF (Gustafsson et al., 2016) was used to image a postsynaptic density marker called Homer 1c at subdiffraction resolution (Dani et al., 2010). Thus, dual color subdiffraction limited images of F-actin and Homer 1c were analyzed to reveal the nanoscale architecture of F-actin cytoskeleton in excitatory synapses. This analysis of branched F-actin network in spines was achieved in two steps. (1) A supervised learning tool, trainable Weka segmentation (TWS; Arganda-Carreras et al., 2017), was used to identify F-actin-enriched regions overlapping with Homer 1c, and a custom designed classifier was used to sort these regions into distinct subsets of spines based on their morphology. (2) A deep neural network (DNN) architecture called artificial neural network accelerated photoactivated localization microscopy (ANNA-PALM), previously developed (Ouyang et al., 2018) to predict linear features (tubular/rod-like), was used to extract actin distribution of these F-actin-enriched compartments. The F-actin distribution thus obtained was analyzed within dendritic spines to distinguish between different morphologic classes of spines. Extraction of F-actin networks from these single synapses permitted us to estimate the cumulative F-actin length, as well as to determine the levels of F-actin in the neck and head of individual spines. The present approach reported in this paper allows the observer to objectively probe morphologic characteristics of spines based on F-actin changes. This method has been validated using PREM images revealing F-actin organization in spines. This supervised learning algorithm was then used to elucidate the differences in the properties of the F-actin network between neuronal cultures from wild-type (WT) and a transgenic mouse model of Alzheimer’s disease.

## Materials and Methods

### Super-resolution data

Single molecule based super-resolution data obtained from primary cortical cultures used for this paper has been obtained from a repository of images from a previously published manuscript (Kommaddi et al., 2018). Mixed sex primary cortical neurons were prepared from postnatal day 0/1 (P0/P1) pups from both WT and APP/PS1(APP_Swe_/PS1ΔE9) mouse, as described previously (Kommaddi et al., 2018).

### Primary neuronal cultures

Mixed sex primary hippocampal cultures were prepared from P0/P1 rat pups (Sprague Dawley) using a similar protocol, as described previously (Kommaddi et al., 2018). The neuronal cultures were fixed at 21 days in vitro (DIV 21) and labeled for F-actin and Homer 1c. All the necessary animal ethics protocols used in this study were obtained by the ethical committee of the institute.

### PREM protocol

PREM was performed as described previously (Svitkina, 2016; Efimova et al., 2017). In brief, dissociated rat embryo hippocampal neurons were cultured in Neurobasal media (Gibco) supplemented with 2% B_27_. At DIV 14–17, neurons were extracted with 1% Triton X-100 in PEM buffer (100 mM Pipes-KOH, pH 6.9, 1 mM MgCl_2_, and 1 mM EGTA) containing 2% polyethylene glycol (molecular weight of 35,000), 2 μM phalloidin, and 10 μM taxol for 3 min at room temperature. Detergent-extracted cells were fixed sequentially with 2% glutaraldehyde in 0.1 M Na-cacodylate buffer (pH 7.3), aqueous 0.1% tannic acid, and aqueous 0.2% uranyl acetate, critical point dried, coated with platinum and carbon, and transferred onto 50 mesh electron microscopic grids. Samples were analyzed using JEM 1011 transmission electron microscope (JEOL USA) operated at 100 kV. Images were captured by ORIUS 832.10W CCD camera (Gatan). PREM images are presented in inverted contrast.

### dSTORM

Primary neuronal cell culture experiments and dSTORM based super-resolution imaging were performed, as explained previously (Kommaddi et al., 2018). The super-resolution images of F-actin were obtained using ThunderSTORM, an ImageJ plugin (Schneider et al., 2012; Ovesný et al., 2014), and/or adapted from the existing repository of data that has been published previously.

### SRRF

SRRF is a collection of analytical methods for super-resolution light microscopy which is available as an ImageJ plugin called NanoJ SRRF (Gustafsson et al., 2016). It is a fluctuation-based method, which overcomes the diffraction barrier by a factor of 2. Images of conventional fluorophores such as GFP and many organic dyes can be analyzed with this method. In this study, we have used NanoJ to generate a subdiffraction image of Homer 1c labeled with Alexa Fluor 532 in dendritic spines.

### Super-resolution simulation (SuReSim)

SuReSim (Venkataramani et al., 2016) was used to simulate resolution matched dSTORM like images from PREM images of the cytoskeleton in spines. For this, segmented 10-nm-thin filaments in PREM images were skeletonized manually and was exported as a *.wimp file at the same sampling as that of the PREM images (1 nm/px). The *.wimp file was later imported into the SuReSim interface for simulating resolution matched dSTORM images from the skeletonized images, with a similar sampling as that of regular reconstructed super-resolution images (20 nm/px). For the creation of resolution matched images, the width of the skeleton was approximated to be 10 nm. The epitope density, i.e., the frequency at which the epitope can be labeled on the skeletonized filament, was given as 0.25 nm^−1^. Labeling efficiency was given as 100% at the best labeling. The on-off cycle to mimic single molecule blinking kinetics was given as 5 × 10^−4^ frames (corresponding to once every 2000 frames). Localization precision was given as 20 nm in line with experimental accuracy obtained for single molecules. In the reconstructed super-resolved images, the localization precision of the single molecules was provided as 20 nm and a sampling size of the final images was given as 20 nm/pixel. These settings are provided in basic settings 1 and 2 in the SuReSim module to generate the final image.

### TWS

TWS is a supervised learning ImageJ plugin for image segmentation (Arganda-Carreras et al., 2017). Based on the heterogeneity of the signal from a microscopy image, the user defines three different classes of signals. Class 1 defines the structure of interest, class 2 defines the background, and class 3 any other signal which does not fall in class 1 or class 2. This information is used to train a classifier to segregate the images into three categories, from which class 1 is used for further processing.

### ANNA-PALM for image analysis

ANNA-PALM is a machine learning-based ImageJ plugin trained to predict correlative structures in super-resolution images (Ouyang et al., 2018). It is based on a pix2pix architecture, which is used to predict correlative structures such as microtubules from a small subset of its localization. We used F-actin super-resolution images in ANNA-PALM to generate tubular structures (referred here as “ridges”) using the tubulin model published previously (Ouyang et al., 2018). We refer to this generalized protocol in our manuscript as a tubular model (Ouyang et al., 2018). We cropped 512 × 512 px^2^ regions in the super-resolution images for this analysis. These images were used for subsequent ridge detection and feature analysis.

### Ridge detection on continuous F-actin networks

Ridge detection is used to find the maxima of a signal in an image by approximating the signal to a range of intensity peaks and valleys. The points corresponding to the maximum intensity were approximated to a line which forms the skeleton of the maximum intensity of structures in any given area. The skeletonized structures of the map of intensity maximum depict the ridges that are detected in the image. In an F-actin super-resolution image, it was used to find the extent of tubular structures. Here, we have used ridge detection plugin from ImageJ (Steger, 1998) to map the maxima of tubular structures of networks detected by ANNA-PALM, indicating the skeleton of ridges of F-actin. to create ridges on the ANNA-PALM images, we have used a sigma of 2.81, and lower and upper thresholds of 0 and 0.83, respectively.

### Expert annotation of spines

An online annotation tool was used to get expert annotations on the putative spines extracted from the binary images. The annotation tool is accessible via the link https://www.robots.ox.ac.uk/~vgg/software/via/via_demo.html.

A total of 1056 spines was extracted from WT rat cultures and annotated into one of the four classes (mushroom, stubby, thin, and forked spines). A spine was considered for further analysis only if at least three out of four annotators gave the same label (Extended Data 1). A total of 762 spines passed this selection criterion, including 254 mushroom spines, 398 stubby spines, 102 thin spines, and eight forked spines. As they were too few, forked spines were discarded from further analysis, bringing the total number of spines to 754. Similar annotation and selection procedures were used for WT mouse neurons (51 mushroom spines, 47 stubby spines, and 11 thin spines for a total of 109 spines) and APP/PS1 mouse neurons (17 mushroom spines, 70 stubby spines, and 18 thin spines for a total of 105 spines) (Extended Data 1).

10.1523/ENEURO.0425-18.2019.ed1 Extended Data 1 On the GitHub repository, there are two folders titled rat and mice. Folder rat has a subfolder xls. Contents of xls are: (1) shape_info.xlsx, (2) class_01.xlsx, (3) class_02.xlsx, (4) class_03.xlsx, and (5) class_04.xlsx. Shape_info.xlsx contains the 22 features identified using Shape Filter plugin in ImageJ class_01.xlsx, class_02.xlsx, class_03.xlsx, and class_04.xlsx contain annotations of spines from four human experts, respectively, of all the 1056 spines. MATLAB code files: shapeinfo_cluster.m, reduces shape information to five dimensions using PCA. These five dimensions are used for training a SVM using MATLAB function *fitecoc* to classify the spines into three categories. get_head_neck_regions.m, computes cumulative branch lengths for head and neck regions separately from the image input. compare_head_neck_len.m, this code plots the lengths of the branches from head and neck regions as a histogram using nhist.m function. The F-actin images of dendritic spines from rat neuronal cultures is in the folder spines.rar. Folder mice has a subfolder xls. Contents of xls are: (1) shape_info_mice.xlsx, (2) class_01.xlsx, (3) class_02.xlsx, (4) class_03.xlsx, and (5) class_04.xlsx. Shape_info_mice.xlsx contain the 22 features identified using Shape Filter plugin in ImageJ class_01.xlsx, class_02.xlsx, class_03.xlsx, and class_04.xlsx contain annotations of spines from four human experts, respectively, of all the 249 spines. MATLAB code files: shapeinfo_cluster.m, reduces shape information to five dimensions using PCA. These five dimensions are used for training a SVM using MATLAB function *fitecoc* to classify the spines into three categories. get_head_neck_regions.m, computes cumulative branch lengths for head and neck regions separately from the image input. compare_head_neck_wt_tg.m, this code plots the lengths of the branches from head and neck regions as a histogram using nhist.m function. cumlen_wt_tg_stubbythin.m, computes cumulative F-actin lengths for stubby and thin. The F-actin images of dendritic spines from mice neuronal cultures are in the folder spines.rar. Download Extended Data 1, ZIP file.

### Principal component analysis (PCA)

The shape filter from ImageJ was used to extract 22 different shape characteristics of the F-actin distribution in dendritic spines from binary images of spines such as area, perimeter, etc. (Wagner and Lipinski, 2013). The 22 shape-based features for 754 and 214 spines from primary neuronal cultures from rat and mouse, respectively, were collected in separate matrices, with each row representing the feature vector for a single spine. Each column of this matrix was normalized by z-scoring and submitted to PCA using the *pca* function in MATLAB (R2015b, academic license). It was found that the first five principal components explained ∼90% of the variance in the original 22-dimensional data. The projection of the 22-dimensional data onto these five principal components was used for further clustering analysis.

### Classification of sines using a linear classifier

A three-way linear support vector machine (SVM) classifier was trained on the principal component representation of 754 spines from rat cultures using the MATLAB function *fitecoc*. To avoid overfitting, a k-fold cross-validation approach was used with k = 4. A slightly different procedure was used to classify spines from mouse cultures. A three-way linear SVM classifier was trained on the principal component representation of 109 WT spines with 4-fold cross-validation. This linear SVM model was then used to classify APP/PS1 spines into mushroom, stubby or thin categories. However, the performance remained comparable even after training the classifier on the combined dataset of 214 WT and APP/PS1 spines.

### Resolution scaled Pearson’s coefficient (RSP) and resolution scaled error (RSE) map

RSP and RSE were determined using the NanoJ SQUIRREL plugin of ImageJ (Culley et al., 2018) with the magnification parameter set as 1 (Venkatachalapathy et al., 2019).

### Software accessibility

All codes and data used for analysis in the paper are made available to the scientific community at the following link: https://github.com/arty-p/auto-factin.git. All MATLAB (R2015b v8.6.0.267246, student license) scripts were run on a computer running Windows 10 pro N (64-bit) operating system with Intel i7-4770 CPU and 32 GB RAM.

### Statistics

We report the mean and the standard deviation for all parameters. However, while calculating the significance levels, we first test for normality and accordingly use *t* test when the distribution is normal, and rank sum test when the distribution is non-normal. All the analyses were performed on the MATLAB.

## Results

### Workflow for morphologic characterization of spines and feature extraction from super-resolution images

**Figure 1.**
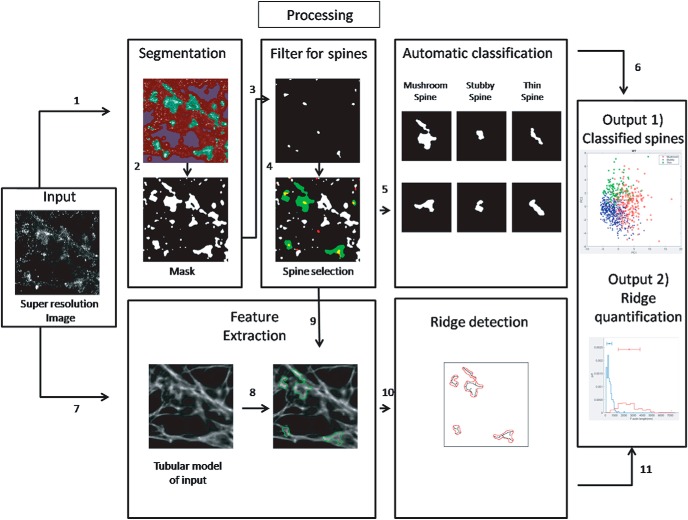
Schematic representation of the workflow for generating an objective classification of F-actin organization in dendritic spines. The super-resolution image of F-actin generated using dSTORM microscopy is considered as the input. (1) Using the TWS on input, a segmented image was created. (2) The segments of interest were color coded and a binary image was obtained for F-actin-enriched regions (mask). (3) The super-resolution image of Homer 1c was generated for the same region of interest as that of input. (4) The segmented image of input was spatially correlated with the postsynaptic marker Homer 1c to select for dendritic spines; please refer to Extended Data Fig. 1-1 for a detailed work flow for steps 1-4. (5) The spines obtained from step 4 were further categorized as mushroom, stubby, and thin using supervised learning. (6) The final data were categorized and plotted into different classes as output 1. (7) The tubular model of the input image was generated using ANNA-PALM. (8, 9) Two processing steps were converged to understand the nanoscale distribution of F-actin in dendritic spines generated from the tubular model, which was spatially correlated with Homer 1c-positive regions obtained in step 4. (10) Spine-specific ridges were extracted in the regions identified positive for excitatory synapses; please refer to Extended Data Fig. 1-2 for a detailed work flow for steps 8-10. (11) The spine-specific parameters of the ridges were measured and plotted as output 2.

dSTORM imaging (20,000 frames at 33 Hz) was performed and super-resolution images of F-actin in primary neuronal cultures immunolabelled with phalloidin-Alexa Fluor 647 were reconstructed. A series of frames (4000 frames at 33 Hz) were captured to record the intensity fluctuations of Alexa Fluor 532-labeled Homer 1c, which was later analyzed by SRRF. A schematic of the workflow for supervised learning based analysis to extract nanoscale features of F-actin from individual dendritic spines is depicted in Figure 1. Super-resolution images of F-actin were processed using TWS and ANNA-PALM in parallel steps to select for F-actin rich regions in neuronal processes, and to create a tubular model of F-actin network, respectively. The super-resolution image of F-actin is considered as the “input.” The SRRF image of Homer 1c, marking the postsynaptic compartment, is referred to as the “reference” (Fig. 1).

The input (Extended Data Fig. 1-1*A*) was treated by TWS to extract F-actin rich compartments from the dSTORM image (Extended Data Fig. 1-1*B*). Here, the user defines three classes of F-actin signals on the image for segmentation. A binary image of the class 1 signal was generated as an outcome of this segmentation and is referred to as the mask (Extended Data Fig. 1-1*C*). The mask represented all the F-actin rich compartments in the neuronal processes (Extended Data Fig. 1-1*C*). Presence of Homer 1c was used to confirm the presence of dendritic spines (Extended Data  Fig. 1-1*D*). To identify the Homer 1c-enriched compartments, the reference image was segmented through TWS. Similar to the input, class 1 signal of the reference was binarized (Extended Data Fig. 1-1*E*). This binarized image is referred to as the filter (Extended Data Fig. 1-1*F*). The filter represented the sites of the postsynaptic density and was used to identify the regions colocalizing with the mask generated from the input image (Extended Data Fig. 1-1*G*). The extracted Homer 1c-positive mask was automatically classified using a supervised learning protocol into different classes of dendritic spines based on their morphologic features, as explained in the following section (Extended Data Fig. 1-1*H*). The classified spines were graphically represented and color coded based on their morphologic identity and is depicted as output 1 (Fig. 1). We verify that the segmented Homer 1c puncta are distributed with a mean area of 0.048 ± 0.024 µm^2^. This value compares with the reported average PSD area of 0.069 µm^2^ (Harris and Weinberg, 2012).

10.1523/ENEURO.0425-18.2019.f1-1 Extended Data Figure 1-1 Feature-based supervised learning approach for structure identification. ***A***, A dSTORM image of F-actin from neuronal culture. ***B***, Feature-based segmentation of the dSTORM signal of F-actin and segregation into class 1 (green), class 2 (purple), and class 3 (red). ***C***, Mask of segmented F-actin signal which contains putative spines. ***D***, SRRF image of the postsynaptic marker Homer 1c. ***E***, Feature-based segmentation of the SRRF signal of Homer 1c and segregation into class 1 (green), class 2 (purple), and class 3 (red). ***F***, Mask of a segmented signal indicating the nanoscale localization of postsynaptic density. Scale bar = 500 nm. ***G***, Colocalization of the mask of segmented F-actin with that of Homer 1c. ***H***, Categorization of F-actin-enriched compartments with PSD as spines (green), which were exported for further shape-based analysis. Scale bar = 500 nm. Download Figure 1-1, EPS file.

In parallel, the input was processed using ANNA-PALM to generate a network of F-actin distribution using the tubular model (Extended Data Fig. 1-2). This tubular model was generated through supervised learning of tubular/rod-like network. This image generated by ANNA-PALM was overlaid with the corresponding mask positive for Homer 1c, marking excitatory synapses (Extended Data Fig. 1-2). The regions of the F-actin network corresponding to individual excitatory synapses were extracted and analyzed according to their morphology. The properties of F-actin network such as the cumulative length of F-actin are plotted as output 2 (Fig. 1).

**Figure 2.**
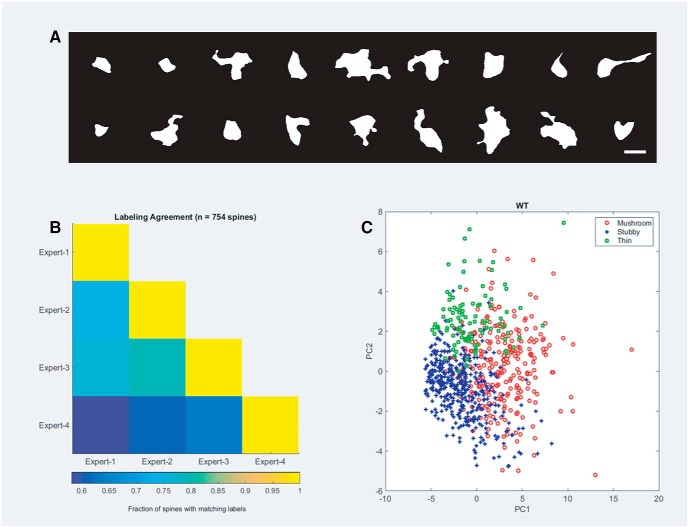
Supervised learning algorithm for morphologic characterization of spines from primary rat hippocampal neurons. ***A***, A gallery of different morphologies of F-actin-enriched compartments in primary rat hippocampal neurons identified as spines. Scale bar = 1 μm. ***B***, A matrix which depicts pair-wise agreement between different experts to classify spines into distinct morphologic classes. The pseudocolor bar depicting the pairwise agreement is shown below. ***C***, A 2-dimensional representation of the classification using two principal components showing that the morphologic characterization of spines forms three nonoverlapping regions. The morphologic features were used for cataloging F-actin structure into a distinct spine category.

10.1523/ENEURO.0425-18.2019.f1-2 Extended Data Figure 1-2 Identifying F-actin organization using ridge detection in single spines. ***A***, Feature-based segmentation of the dSTORM signal of F-actin and segregation into class 1 (green), class 2 (purple), and class 3 (red). ***B***, The input dSTORM images were transformed into the tubular model using ANNA-PALM. ***C***, The ANNA-PALM image was transformed and skeletonized using ridge detection module to represent the F-actin ridges. ***D***, The segmented regions colocalizing with the postsynaptic marker Homer 1c were extracted. ***E***, The F-actin mask was used to selectively filter spine-specific F-actin ridges (black) in ***C***. ***F***, The selected ridges (black) depicted bundled F-actin within each spine (red). Scale bar = 500 nm. Download Figure 1-2, EPS file.

### Classification of spines into different morphologic classes using supervised learning

After identifying F-actin masks which were positive for dendritic spines, we developed an automated tool based on supervised learning for morphologic characterization of dendritic spines (as mushroom, stubby or thin), which has never been performed on dSTORM images. For the purpose, we computed 22 shape-based features (such as area, perimeter, aspect ratio, etc.) using the Shape Filter ImageJ plug-in for 754 spines from primary rat hippocampal cultures. We reduced the dimensionality of this feature representation to five dimensions using PCA to classify spines from the dSTORM data (Fig. 2*A*). These five dimensions captured ∼90% of the variance in the data. We trained an SVM classifier on these five dimensions and sorted the spines into three different categories. The agreement between human experts is presented in Figure 2*B*. The trained classifiers had an accuracy of 82.6% (on 754 spines with 4-fold cross-validation) compared to the performance by the human experts. The graphical representation of PCA after supervised learning was color coded for different morphologic classes of spines (Fig. 2*C*).

### Extraction and validation of branched F-actin networks from dendritic spines

To approximate the F-actin network (Extended Data Fig. 1-2*A*) to a tubular/rod-like distribution, we used ANNA-PALM to generate a tubular network model on dSTORM images of F-actin (Extended Data Fig. 1-2*B*). This gave a continuous network architecture for F-actin in neuronal processes, which was limited by the resolution of our experimental system. We performed the ridge detection analysis to identify the distribution of F-actin rods in the ANNA-PALM image (Extended Data Fig. 1-2*C*). The ridges were detected in all regions where the F-actin network could be resolved (Extended Data Fig. 1-2*C*). to analyze the distribution of F-actin in individual spines (Extended Data Fig. 1-2*D*), synapse-specific ridges were extracted from the mask of F-actin-rich regions overlapping with the postsynaptic marker Homer 1c (Extended Data Fig. 1-2*E*). Branched F-actin distribution was isolated based on the morphology of individual spines, as indicated in the previous section (Extended Data Fig. 1-2*F*).

**Figure 3.**
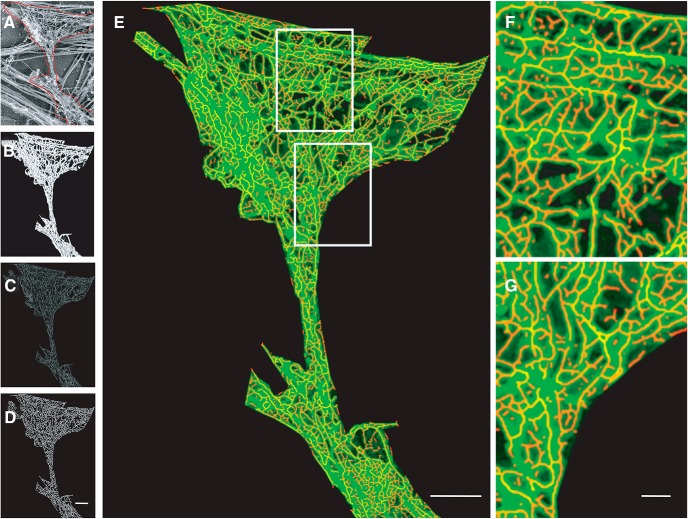
Analysis of nano-organization of F-actin at 1-nm/px sampling. ***A***, PREM image of cytoskeletal distribution within a spine. Scale bar = 200 nm. ***B***, The segmented image selecting only the thin filaments in PREM indicate the F-actin distribution. ***C***, ANNA-PALM simulation of the F-actin network using tubular model. ***D***, Extraction of ridges by skeletonizing the ANNA-PALM image. ***E***, Overlay of an image obtained by PREM (green) and ridges that mark the F-actin network (red) of the spine. Scale bar = 200 nm. ***F***, ***G***, Magnified views of sections within the spine; please refer to Extended Data Fig. 3-1 for a comparative analysis between PREM and simulated super resolved images. The ridges overlapped with the PREM images with a correlation of >89%. Scale bar = 50 nm.

The ridges extracted from dendritic spines of super-resolution images of F-actin presented a highly branched structure, which was variable between spines (Extended Data Fig. 1-2*F*). We evaluated whether this structure was indeed present in spines or whether it was an artifact of dSTORM imaging. We verified this using PREM images of F-actin obtained from rat hippocampal neurons (Fig. 3*A*; Extended Data Fig. 3-1*A–C*; Efimova et al., 2017). The sampling for super-resolution images obtained by dSTORM was 20 nm/px, while that obtained from PREM was 1 nm/px. The PREM images were a mix of different kinds of filamentous structures that are observed inside neurons (Fig. 3*A*). However, only the filaments which were smaller than 10 nm including the platinum layer represented F-actin. To overcome the sampling difference, we extracted exclusively F-actin thin filaments (<10 nm) from PREM images using TWS and used ANNA-PALM to fit the segmented image by a tubular model (Fig. 3*B*,*C*). The ridge detection module was then applied to identify the skeleton of this distribution, which we refer to as ridges (Fig. 3*D*). We found that the ridges overlapped with the F-actin network with a correlation of 0.89 (Fig. 3*E*, insets 1, 2), indicating that F-actin in spines could be fit with the tubular model and the detected ridges represented the skeleton of the F-actin network in spines.

10.1523/ENEURO.0425-18.2019.f3-1 Extended Data Figure 3-1 Simulation of dSTORM like images of F-actin from PREM images. ***A–C***, Examples of PREM images with 1-nm/px sampling of a subsection of a neuronal process, where the red region indicates the presence of a spine. ***D***–***F***, Simulation of single molecule based super-resolution images using SuReSim, with 20-nm/px sampling, of F-actin cytoskeleton in spines identified by PREM. ***G–I***, Approximation of tubular rod-like distribution of F-actin nanoscale images using ANNA-PALM. ***J–L***, Error of mismatch between the tubular model and the simulated single molecule based super-resolution image. The mean RSP between the model and the simulated dSTORM image was 0.89 ± 0.03. The pseudocolor bar ranging from purple to yellow indicates low to high error. Scale bar = 200 nm Download Figure 3-1, EPS file.

At 20-nm/px sampling, the dense network of F-actin was undersampled, resulting in loss of resolution of F-actin features. The difference in the lateral resolution between a PREM image (1 nm/px, resolution 2.5 nm) and a dSTORM image (20 nm/px, 40–45 nm) is 16–20 times. Using SuReSim, we simulated a dSTORM image of the PREM image to mimic the loss of resolution (Extended Data Fig. 3-1*D–F*). We performed ANNA-PALM on the simulated image to verify the cumulative content of F-actin after ridge detection. The cumulative length of F-actin from ridges was 64.8–71.7 µm at 1 nm/pixel in contrast to 4.3–6.6 μm at 20 nm/px. This suggested that despite a resolution difference of 20 times between PREM and super-resolution light microscopy, the average change in the detected ridges of F-actin was only 10- to 12-fold. This indicated that although the same PREM data sampled at different resolutions provided reduced information, this reduction was much less compared to the change in resolution between these regimes. Interestingly, super-resolution experiments in primary rat hippocampal neurons estimated the cumulative F-actin content in mushroom spines to be 4–6 µm (data not shown), corresponding well with the range predicted by the simulated experiments above. This confirmed that the resolution was consistent between simulation and experiment, validating the robustness of dSTORM despite its lower resolution compared to PREM. Furthermore, when we compared the simulated dSTORM image at 20nm/px to its corresponding tubular model of F-actin network, the RSP was 0.89 (Extended Data Fig. 3-1*K–M*), indicating a high correlation between experimentally observed dSTORM images and their corresponding tubular model (0.90). This correlation between simulation and the experiment reiterated the validity of dSTORM in extracting branched network features of F-actin through a combination of ANNA-PALM and ridge detection.

**Figure 4.**
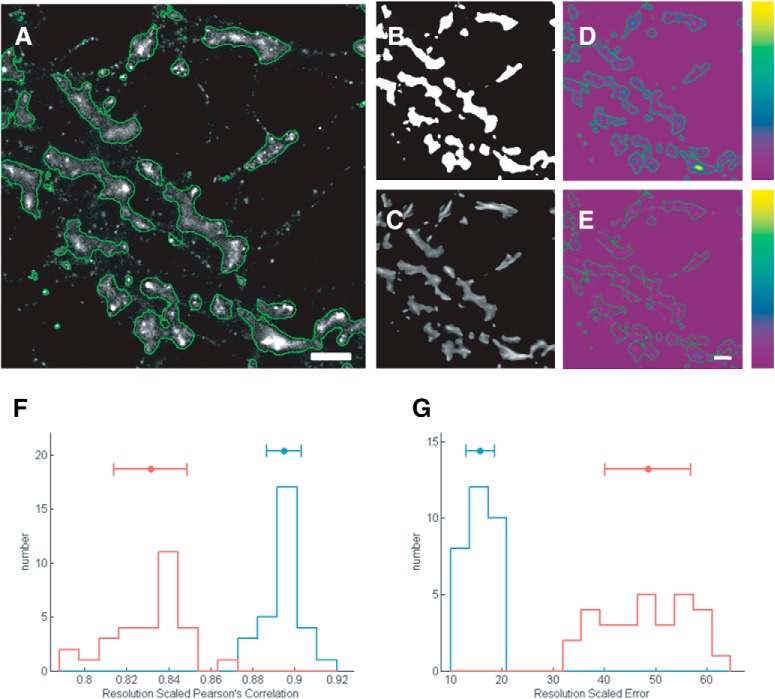
Tubular model of F-actin represents its actual distribution in spines. ***A***, Super-resolution image of F-actin in neurons obtained by dSTORM. Scale bar = 1 μm. ***B***, Mask of F-actin rich compartments in neuronal processes. ***C***, Tubular model of F-actin obtained by ANNA-PALM. ***D***, RSE maps indicating the correlation between dSTORM image and F-actin mask. ***E***, RSE maps of the dSTORM image with a tubular model of F-actin. Scale bar = 1 µm. The pseudocolor bar ranging from purple to yellow indicates low to high error. ***G***, RSP of dSTORM image with the F-actin mask (red) and with the tubular model of F-actin from ANNA-PALM (blue). ***I***, RSE of the dSTORM image with the F-actin mask (red) and with the tubular model of F-actin from ANNA-PALM (blue).

To further validate the robustness of our data, super-resolution images were acquired from neurons co-labeled with phalloidin-Alexa Fluor 647 (dSTORM) and Homer 1c (Alexa Fluor 532). The localization precision of the experimental system generated was 19 nm, with a sampling of 20 nm/px (similar to simulated dSTORM images), and the final experimental resolution of the image was calculated to be 44 nm/px (Kommaddi et al., 2018). Similar to the analysis performed for the PREM images, we quantified the extent of mismatch between the tubular model and the dSTORM super-resolution image (Fig. 4). For this, we calculated the RSP (Fig. 4*D*) and RSE (Fig. 4*E*) between the original super-resolution image of F-actin corresponding to the dendritic spines extracted through TWS segmentation, and the tubular model obtained by ANNA-PALM, respectively (Fig. 4*A–C*). We found that RSP of super-resolution image of F-actin with either the tubular model or the mask obtained through TWS was above 0.90, indicating a good correlation. On evaluating the RSE, the ANNA-PALM modeling showed the least error with the dSTORM data, indicating a good fit between the network model and super-resolution image, further validating the robustness of the analysis in the experimental conditions (Fig. 4).

### Quantification of F-actin architecture in dendritic spines of primary cortical neuronal cultures derived from the transgenic mouse model of AD

**Figure 5.**
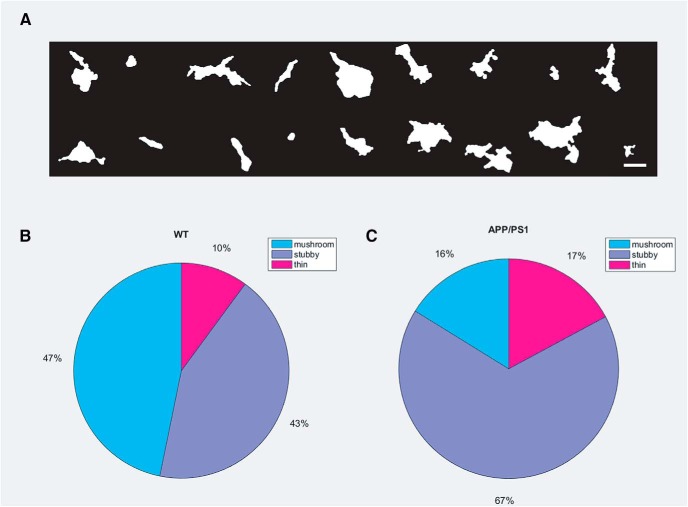
Comparison of morphologic features of spines obtained by supervised learning algorithm from WT and APP/PS1 primary mice cortical neurons. ***A***, A gallery of different morphologies of F-actin-enriched compartments in primary mice cortical cultures identified as spines. Scale bar = 1 μm; please refer to Extended Data Fig. 5-1 for the validation of supervised learning algorithm for morphological characterization of spines from primary mice cortical neurons. ***B***, A pie-chart representing proportion of mushroom, stubby and thin spines in WT. ***C***, A pie-chart representing proportion of mushroom, stubby and thin spines in the entire population of dendritic spines in APP/PS1; please refer to Extended Data Fig. 5-2 to view a set of morphologies of spines characterized as mushroom and thin spines.

Using the supervised learning classification method established previously in rat primary hippocampal neurons, we investigated F-actin distribution in dendritic spines of primary cortical neurons of WT mice (Fig. 5*A*). The labeling of spines was obtained through expert human annotations with the pairwise agreement of 88% (Extended Data Fig. 5-1*A*). Further, the linear SVM classifier reached an accuracy of 86.2% for the same, with 4-fold cross-validation (Extended Data Fig. 5-1*A*). The mask of super-resolution images of dendritic spines was a better marker for their morphology. It was possible to classify mushroom spines with shorter necks and oddly shaped thin spines with intricate morphologies which would otherwise have fallen into the category of stubby spines if acquired by conventional light microscopy (Extended Data Fig. 5-2). We applied the same analysis for spines obtained from cultures of transgenic mice (APP_Swe_/PS1ΔE9 [APP/PS1]) encoding genetic mutations in Amyloid Precursor Protein (APP) and Presenilin 1 (PS1). This enabled a direct comparison of spine shapes based on F-actin content across healthy and diseased conditions. This automated classification showed a reduction of mushroom spines from 47% to 16%, and a corresponding increase in both stubby and thin spines from 43% and 10% to 67% and 17%, respectively, from WT to transgenic mice (Fig. 5*B*,*C*).

10.1523/ENEURO.0425-18.2019.f5-1 Extended Data Figure 5-1 Supervised learning algorithm for morphological characterization of spines from primary mice cortical neurons. ***A***, A matrix which depicts pair-wise agreement between different experts to classify spines into distinct morphological classes. The pseudocolor bar depicting the pairwise agreement is shown below. ***B***, A 2-dimensional representation of the classification using two principal components showing that the morphological characterization of spines forms three non-overlapping regions. A 2-dimensional representation of the classification using two principal components shows that there exist three categories of spines in both WT and APP/PS1, and thus can be used for predicting if a given structure belongs to any of the three categories (red o, WT mushroom; maroon o, APP/PS1 mushroom; dark blue +, WT stubby; light blue +, APP/PS1 stubby; dark green o, WT thin; light green o, APP/PS1 thin). Download Figure 5-1, EPS file.

10.1523/ENEURO.0425-18.2019.f5-2 Extended Data Figure 5-2 A gallery of super-resolution images of mushroom and thin spines. The top panel depicts mushroom spines with very short necks, which would be classified as a different morphological entity by conventional light microscopy. The bottom panel depicts oddly oriented thin spines, which would be characterized as stubby spines by conventional microscopy. Scale bar = 500 nm Download Figure 5-2, EPS file.

**Figure 6.**
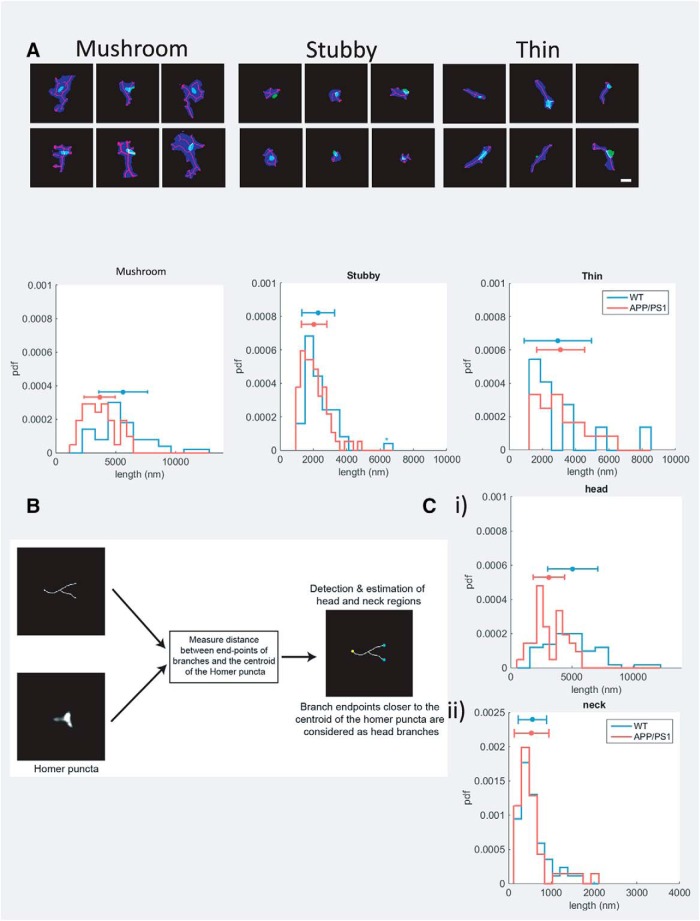
Objective paradigm for segmentation and feature detection in dendritic spines. ***A***, Representative gallery of different classes of dendritic spines are depicted with each class containing six representative spines. Scale bar = 500 nm. We found that the cumulative length of F-actin filaments in mushroom spines were significantly higher in WT spines compared to APP/PS1 spines (average actin filament length: WT mushroom = 5634.5 ± 2034 nm; and APP/PS1 mushroom = 3665.1 ± 1299.2 nm; *p* < 0.005 for a rank sum test on cumulative F-actin filament lengths for individual spines of WT and APP/PS1 groups), while there was no significant difference in the lengths of the F-actin networks in stubby and thin spines (WT stubby = 2288.5 ± 982.6 nm; APP/PS1 stubby = 2045.4 ± 763.9 nm; WT thin = 2927.3 ± 2023.5 nm; APP/PS1 thin = 3098.9 ± 1439.9 nm; *p* = 0.12 and *p* = 0.42 for a rank sum test on cumulative F-actin lengths of stubby and thin spines, respectively). ***B***, The paradigm for feature extraction was performed in two steps. (1) The branch endpoints of the detected ridge of the spine were compared to the centroid of the Homer puncta to define the neck (yellow) and head regions (cyan) of the spine. (2) The length of the ridges was plotted for analysis. ***B***, The difference in the cumulative F-actin filament lengths in mushroom spines was due to difference in their lengths in the head region, rather than the neck (average actin filament length: 5075.7 ± 2048.6 nm and 3126.2 ± 1284.3 nm for WT and APP/PS1 head regions, respectively, *p* < 0.005 for a rank sum test; 558.7 ± 331.7 and 538.9 ± 404.5 nm for WT and APP/PS1 neck regions, respectively, *p* = 0.31 for a rank sum test).

The previous report had shown specific differences in the cumulative length of F-actin in WT and APP/PS1 spines. Here, we validated our analysis paradigm by replicating this result. We first classified the cumulative length of branched F-actin based on different spine morphologies (Fig. 6*A*). The average cumulative length of F-actin in the mushroom spines of WT and the APP/PS1 cultures were 5634.5 ± 2034 and 3665.1 ± 1299.2 nm, respectively. On the other hand, stubby and thin spines displayed a negligible change from 2288.5 ± 982.6 and 2927.3 ± 2023.5 nm in WT conditions to 2045.4 ± 763.9 nm and 3098.9 ± 1439.9 nm in APP/PS1 cultures, respectively. Since the cumulative F-actin content of mushroom spines from WT and APP/PS1 mice showed a significant difference in contrast to the other spine classes, the former was selected for further investigation (Table 1). We then explored whether the reduction of F-actin in the mushroom spines were predominantly from the spine head or from the neck. For this, we used an additional classification to spatially annotate the spine head and the neck (Fig. 6*B*). The branch points of the F-actin filaments closer to the centroid of the Homer 1c staining was denoted as the endpoint for the actin branches in the head, while the farthest endpoint of the actin filament from the Homer 1c was denoted as the endpoint of the spine neck. This procedure enabled us to extract cumulative F-actin lengths from the head and neck regions of the spine. Head region showed a significant reduction of cumulative length of F-actin from 5075.7 ± 2048.6 nm in WT to 3126.2 ± 1284.3 nm in APP/PS1, while in the neck region the values remained unaltered (Table 1; Fig. 6*C*).

**Table 1.** Cumulative length of F-actin in spines and subspine compartments

		Cumulative length of F-actin (nm)		
Spine type	Subspine compartment	WT	APP/PS1	Significance
Mushroom	-	5634.5 ± 2034	3665.1 ± 1299.2	<0.005, yes
	Spine head	5075.7 ± 2048.6	3126.2 ± 1284.3	<0.005, yes
	Spine neck	558.7 ± 331.7	538.9 ± 404.5	0.31, no
Stubby	-	2288.5 ± 982.6	2045.4 ± 763.9	0.12, no
Thin	-	2927.3 ± 2023.5	3098.9 ± 1439.9	0.42, no

Our results match well with the subjective evaluation of F-actin distribution reported previously (Kommaddi et al., 2018), which presented only the cumulative length of F-actin from mushroom spines. In addition to the F-actin distribution, we have presented an automated morphologic classifier which separated the spines using shape-based-features. This morphologic classifier enabled us to separate the F-actin distribution in mushroom, thin and stubby spines. Furthermore, we were able to extract the cumulative F-actin length from subspine compartments like spine head and spine neck, which was also not reported earlier. We show that the objective paradigm that we present in the manuscript describes an unbiased quantification of nanoscale organization of F-actin from individual spines, which can be used to analyze large datasets.

## Discussion

Because of a growing need to analyze the role of F-actin cytoskeleton in morpho-functional changes in spines, automated analysis is required to obtain an objective measure of changes in F-actin organization at the level of individual synapses. Although super-resolution imaging (20–150 nm) is routinely used in many laboratories, most of the morphologic characterization of spines is still performed using volume markers and conventional microscopy, either alone or co-labeled with synaptic markers. Thin spines with complex orientation or mushroom spines with shorter neck could also be mislabeled when imaged by a conventional light microscope. This argues for a need to acquire super-resolution light microscopy images to increase the accuracy of shape-based classification of spines (Tønnesen et al., 2014; Bartol et al., 2015; Kasthuri et al., 2015). Here, we explain a user guided objective protocol whose results are comparable to subjective analysis. This paradigm is automated and can be used for high throughput analysis, thus making it efficient and reproducible. We have illustrated this using data of spines from primary hippocampal (rat) and cortical (mice) cultures co-labeled for F-actin and the postsynaptic marker Homer 1c. We have also compared differences in the F-actin distribution in individual synapses between WT and a transgenic model for Alzheimer’s disease (APP/PS1). A key feature of this automated paradigm is its ability to extend the morphologic classes to include stubby and thin spines in super-resolution images. This has enabled us to classify spines in neurons under different conditions, which was difficult with conventional light microscopy. We show that in primary cortical cultures of WT versus transgenic, the predominant effect on the cumulative length of F-actin was observed in mushroom spines, while the same in stubby and thin spines remain unaltered. In addition, the paradigm enabled quantification of F-actin length from subspine compartments such as head and neck, where there was a significant reduction of cumulative F-actin in the spine head. In the transgenic, there was also a notable reduction in the proportion of mushroom spines with a corresponding increase in stubby and thin spines. This validates the previous observation in hippocampal slices, where there was an augmentation of stubby spines in the transgenic mouse model for Alzheimer's disease in comparison to the control. However, those experiments performed on hippocampal slices were from three-month-old animals (Androuin et al., 2018), while the effect observed in this work is at a much earlier stage as DIV 21. This indicated that besides a large change in morphologic features of spines, the major regulation of F-actin during early stages of Alzheimer's disease occurs predominantly in the head region of mushroom spines.

Automated spine classification by supervised learning has been recently used to classify spines imaged by conventional light microscopy (Zhang et al., 2007; Ghani et al., 2017). Here, we show that by exploiting F-actin dSTORM signal in primary neuronal cultures, the supervised learning approach can also be extended to any subdiffraction limited image. In the future, attempts could be made to use predictive tools to guess how F-actin network organization would appear at electron microscopic resolution using super-resolution images as input (Ouyang and Zimmer, 2017). This would imply that the F-actin characteristics that we define could be improved at an even better resolution. Here, we present significant differences in F-actin organization in subsets of spines in different conditions. Future experiments combining correlative electron microscopy and 3-dimensional super-resolution light microscopy would be optimal to confirm these results, which is beyond the scope of the present study.

In the present work, we have quantified changes in the branched F-actin network in spines and evaluated some of the early changes predicted to occur during the onset of AD. Most of the neurodegenerative diseases, genetic disorders and changes in the strength of the synapses are correlated with changes in spine morphology and F-actin organization. Thus, it is interesting to see whether this paradigm could be used as a common resource to analyze large datasets that can be obtained for different conditions. It remains to be seen whether the same model of analysis could also be used for understanding branched F-actin networks in the growth cone, axonal boutons and inhibitory synapses.

## Conclusion

The supervised learning protocols as predictive models in well-characterized systems is an efficient tool for high throughput analysis of the nanoscale organization. In the present case, using supervised learning along with effective segmentation strategies, we have characterized both morphologies of spines and nanoscale organization of F-actin cytoskeleton. Future work may focus on acquiring and analyzing F-actin structures in spines in 3-dimension at an improved resolution to allow more accurate identification of changes accompanying plasticity or neurodegenerative diseases.

## Synthesis

Reviewing Editor: Alfonso Araque, University of Minnesota Decisions are customarily a result of the Reviewing Editor and the peer reviewers coming together and discussing their recommendations until a consensus is reached. When revisions are invited, a fact-based synthesis statement explaining their decision and outlining what is needed to prepare a revision will be listed below. The following reviewer(s) agreed to reveal their identity: Rácz Bence.

Both reviewers are largely satisfied with the responses and the revised manuscript.

However, there are comments few minor issues that are required to be addressed. Addressing these concerns will enhance the strength of the manuscript.

I am looking forward to receiving a revised version of the manuscript.

Sincerely

Specific comments of the reviewers:

Reviewer 1.

The manuscript is much improved. I have these remaining minor comments:

*In the Introduction (lines 59-60), they make it sound like volume labels like GFP are problematic for ‘health and biophysical properties of the cell’. Why do they say that? GFP is the most commonly used volume label on the planet. Conjugating dyes to proteins of interest or creating fusion constructs, that's problematic because of forced over-expression and potential effects on the protein, which is inevitably the case when trying to label F-actin.

*Authors should consider citing Frost et al (Neuron 2010) and Urban et al (Biophys J, 2011), which are the first studies that imaged actin organization inside live spines with super-resolution (single molecule and STED, respectively).

*Fix spelling mistake in a few places: Principal Component Analysis, not ‘principle’

Reviewer 2.

The revised manuscript presents a novel and robust method/tool to study actin-rich spines with superresolution microscopy and detect changes in actin organization in disease models.

Authors substantially revised the original manuscript addressing all of my concerns in details. I still think that the presented method will not likely be the golden standard

for spine-actin analysis. Nonetheless, the breadth and ambitious attempt of the study to characterize changes in actin organization in a disease model makes the study suitable for publication.

There are no further comments.

## Author Response

Both reviewers are largely satisfied with the responses and the revised manuscript.

However, there are comments few minor issues that are required to be addressed. Addressing these concerns will enhance the strength of the manuscript.

We are grateful to the editor and the reviewers for evaluating the manuscript favourably for publication. We have addressed all the concerns indicated by the reviewers.

Specific comments of the reviewers:

Reviewer 1.

The manuscript is much improved. I have these remaining minor comments:

We thank the reviewer for finding merit in the manuscript. We have addressed all the concerns addressed by the reviewer. Please find the responses below.

*In the Introduction (lines 59-60), they make it sound like volume labels like GFP are problematic for ‘health and biophysical properties of the cell’. Why do they say that? GFP is the most commonly used volume label on the planet. Conjugating dyes to proteins of interest or creating fusion constructs, that's problematic because of forced over-expression and potential effects on the protein, which is inevitably the case when trying to label F-actin.

As suggested by the reviewer, we have changed the sentence to “Conjugating dyes to proteins of interest or creating fusion constructs can create undesirable effects due to excessive expression and steric interference with protein functions”. This change is indicated in red in the section Introduction, paragraph 2, lines 7-9 in the revised manuscript.

*Authors should consider citing Frost et al (Neuron 2010) and Urban et al (Biophys J, 2011), which are the first studies that imaged actin organization inside live spines with super-resolution (single molecule and STED, respectively).

We have included the references suggested by the reviewer in the revised manuscript in the section Introduction, paragraph 1, lines 9-10.

*Fix spelling mistake in a few places: Principal Component Analysis, not ‘principle’

We are grateful to the reviewer for pointing this error. These mistakes, previously in the section Figure Legends, have been corrected and colour coded in the revised manuscript.

Reviewer 2.

The revised manuscript presents a novel and robust method/tool to study actin-rich spines with superresolution microscopy and detect changes in actin organization in disease models. Authors substantially revised the original manuscript addressing all of my concerns in details. I still think that the presented method will not likely be the golden standard

for spine-actin analysis. Nonetheless, the breadth and ambitious attempt of the study to characterize changes in actin organization in a disease model makes the study suitable for publication. There are no further comments.

We thank the reviewer for finding merit in the manuscript.
